# Rapid Reduction in Breast Cancer Mortality With Inorganic Arsenic in Drinking Water

**DOI:** 10.1016/j.ebiom.2014.10.005

**Published:** 2014-10-13

**Authors:** Allan H. Smith, Guillermo Marshall, Yan Yuan, Craig Steinmaus, Jane Liaw, Martyn T. Smith, Lily Wood, Marissa Heirich, Rebecca M. Fritzemeier, Mark D. Pegram, Catterina Ferreccio

**Affiliations:** a Arsenic Research Group, School of Public Health, University of California, Berkeley, Berkeley, CA, United States; b Departamento de Estadística, Facultad de Matemáticas, Pontificia Universidad Catòlica de Chile, Santiago, Chile; c Environmental Health Sciences, School of Public Health, University of California, Berkeley, Berkeley, CA, United States; d Breast Cancer Oncology Program, Stanford Cancer Institute, Stanford, CA, United States; e Departamento de Salud Pública, Escuela de Medicina, Pontificia Universidad Catòlica de Chile; Advanced Center for Chronic Diseases ACCDIS, Santiago, Chile

**Keywords:** Arsenic, Breast cancer, Breast cancer cell line studies, Cancer therapy, Chile, Drinking water, Epidemiology

## Abstract

**Background:**

Arsenic trioxide is effective in treating promyelocytic leukemia, and laboratory studies demonstrate that arsenic trioxide causes apoptosis of human breast cancer cells. Region II in northern Chile experienced very high concentrations of inorganic arsenic in drinking water, especially in the main city Antofagasta from 1958 until an arsenic removal plant was installed in 1970.

**Methods:**

We investigated breast cancer mortality from 1950 to 2010 among women in Region II compared to Region V, which had low arsenic water concentrations. We conducted studies on human breast cancer cell lines and compared arsenic exposure in Antofagasta with concentrations inducing apoptosis in laboratory studies.

**Findings:**

Before 1958, breast cancer mortality rates were similar, but in 1958–1970 the rates in Region II were half those in Region V (rate ratio RR = 0.51, 95% CI 0.40–0.66; p < 0.0001). Women under the age of 60 experienced a 70% reduction in breast cancer mortality during 1965–1970 (RR = 0.30, 0.17–0.54; p < 0.0001). Breast cancer cell culture studies showed apoptosis at arsenic concentrations close to those estimated to have occurred in people in Region II.

**Interpretation:**

We found biologically plausible major reductions in breast cancer mortality during high exposure to inorganic arsenic in drinking water which could not be attributed to bias or confounding. We recommend clinical trial assessment of inorganic arsenic in the treatment of advanced breast cancer.

## Highlights

• We report major reductions in breast cancer mortality during high exposure to inorganic arsenic in drinking water.
• Our breast cancer cell studies showed apoptosis at low concentrations of arsenic.
• We recommend clinical trial assessment of inorganic arsenic in the treatment of advanced breast cancer.

## 1 Introduction

Arsenic has been used therapeutically for more than 2400 years for many different conditions (Antman, 2001). Following initial studies in China in the early 1980s (Zhou, 2012, Wang and Chen, 2008), arsenic trioxide—along with retinoic acid—has become the treatment of choice for relapsed promyelocytic leukemia, usually resulting in complete remission of this highly fatal rare disease (Soignet et al., 1998, Gallagher, 1998). Apoptosis occurs in human promyelocytic leukemia cells exposed to arsenic trioxide in laboratory studies (Iriyama et al., 2013). The idea that human breast cancer might also be treatable with arsenic arose from findings of apoptosis in human breast cancer cells exposed to arsenic trioxide (Xia et al., 2012, Wang et al., 2011, Du et al., 2012, Li et al., 2004, Chow et al., 2004). Several mechanisms have been proposed. For example, arsenic has been shown to cause induction of functional re-expression of the estrogen receptor in estrogen negative breast cancer cells, which could make them less aggressive (Du et al., 2012). Breast cancer remains a major cause of death in the United States and other countries. Mortality has reduced in the last few decades, but less so for estrogen-negative cancers (Jatoi et al., 2007).

Chile is divided into regions, and Region II in northern Chile is extremely dry (the driest inhabited location on earth), with no private wells or alternative water sources, so the entire population uses municipal water. Before 1958, the water of the major city of Antofagasta contained about 90 μg/L arsenic. In 1958, a new system was installed which involved piping water from distant river sources; between 1958 and 1970, arsenic water concentrations averaged 870 μg/L (Marshall et al., 2007). In 1970, an arsenic removal plant was installed and arsenic levels dropped sharply to about 110 μg/L. Arsenic removal has improved and the city water now contains less than 10 μg/L, which is the World Health Organization recommendation for maximum arsenic concentrations in drinking water (World Health Organization, 2011). Other smaller cities and towns in Region II also had high arsenic concentrations during overlapping periods, although not as high as in Antofagasta (Smith et al., 1998).

We have previously demonstrated markedly increased mortality in Region II of Chile from cancers of the lung, bladder, and kidney, starting about ten years after high exposure commenced, with increased risks continuing decades after exposure reduction (Marshall et al., 2007, Smith et al., 1998, Steinmaus et al., 2013, Yuan et al., 2010). These findings are consistent with other studies, and arsenic in drinking water is now an established cause of lung and bladder cancers (International Agency for Research on Cancer, 2004). However, in assessing all causes of death from 1950 to 2010, we were surprised to see that breast cancer mortality was actually reduced during the high exposure period. This paper reports these findings in light of the potential therapeutic role of inorganic arsenic.

## 2 Methods

Computerized mortality data were available for all of Chile after 1970, but not before. We therefore designed a mortality study comparing death rates in Region II with Region V from 1950 to 2010, since it would have been prohibitively expensive to code death certificates before 1970 for the whole country. For the years 1950–1970, we digitally photographed all death certificates (218,000) for Region II and Region V; they were then coded by trained nosologists according to the International Classification of Diseases, Ninth Revision (ICD-9). Death certificate data from both regions were intermingled after computer entry, and nosologists were kept blind as to the region from which death certificates came. After 1970, coded computerized mortality data were obtained from the Instituto Nacional de Estadística de Chile.

### 2.1 Arsenic Exposure Data

**Fig. 1:**
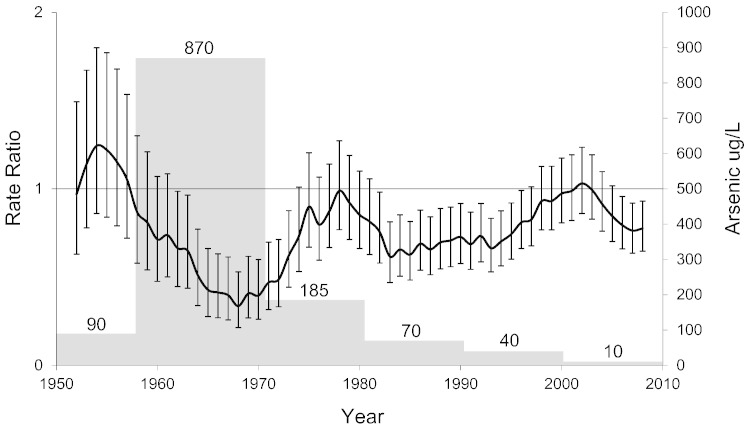
Age-adjusted breast cancer rate ratios and 95% confidence intervals, comparing Region II with Region V, 1950–2010. Each point represents an estimate for 5 years and is plotted at the midpoint of the 5-year period, starting with the estimate for 1950–1954, which is plotted at the year 1952. Gray histograms show arsenic concentration in the drinking water in Antofagasta.

We obtained water arsenic concentrations for Region II of Chile (Smith et al., 1998, Ferreccio et al., 2000), and concentrations for the city of Antofagasta are plotted in Fig. 1. The nearby town of Mejillones shared the same water source, so that about 50% of the population of Region II would have experienced these very high exposures. We also obtained arsenic water concentrations for other cities and towns in Region II, most of which also had increased arsenic concentrations in their water. For example, the second largest city of Calama with a population of 100,283 had arsenic water concentrations above 200 μg/L until 1978, when arsenic removal commenced in that city (Smith et al., 1998).

### 2.2 Comparison Population

We selected Region V of Chile as a comparison population because it was considerably larger than Region II, giving good statistical power, and had never experienced significant population exposure to arsenic in drinking water. For example, the major city of this region (Valparaíso, with a population of about 300,000 in 1970) has arsenic water concentrations close to 1 μg/L (Yuan et al., 2010). A survey of Valparaíso urine arsenic concentrations had an average of 15 μg/L in 1984 (Venturino, 1991), a level typically found in low exposure populations. To check that Region V was an appropriate comparison population with regard to other characteristics, we compared census information for the years 1952, 1960 and 1970.

### 2.3 Statistical Analysis

To investigate the temporal relation of breast cancer and ovarian cancer mortality to changes in the water concentration of arsenic, we categorized calendar years into four time periods on the basis of exposure in Region II: 1950–1957 (low exposure), 1958–1970 (high exposure), 1971–2000 (low-moderate exposure), and 2001–2010 (low exposure). We estimated breast cancer (ICD-9 code 174) and ovarian cancer (ICD-9 code 183) mortality rate ratios (RRs) using Poisson regression analysis comparing Region II with Region V, with adjustment for age in 10-year strata from 30 to 80 years of age (SAS version 9.3, SAS Institute, Inc., Cary, NC).

### 2.4 Breast Cancer Cell Line Studies and Arsenic Concentrations

We conducted studies to investigate the toxic effects of arsenic trioxide on human cancer cells in vitro, using the breast cancer cell lines MCF7, MDA-MB-468, BT474, and SKBR3, and the noncancerous breast cell line MCF10A. Following 72 h of treatment with As_2_O_3_ at varying concentrations between 0.1 and 40 μM, breast cancer cell viability was measured. Breast cancer cells were also treated with 0.5, or 4.5 μM As_2_O_3_ for 72 h, and assessed by immunoblot analysis. Methodological details are given in Supplementary Materials p 1.

## 3 Results

Table 1 presents the census demographic data available for 1952, 1960, and 1970. There were higher percentages of men, consistent with extensive mining activities in Region II. Other factors, including the percentage of births to mothers under the age of 20, were similar for both regions. More recent census data and surveys comparing Region II with Region V or all of Chile (Smith et al., 2012) are summarized in Supplementary Materials p 2. No major differences have been apparent in any factors that might have a meaningful impact on breast cancer mortality.

Table 2 presents breast cancer mortality RRs separated into the main exposure periods (Supplementary Materials p 3 gives more detailed age-group data). Before 1958, breast cancer mortality was about the same in Regions II and V (mortality rate ratio RR = 1.07, 95% CI 0.78–1.47). Breast cancer mortality reduced in Region II in the period 1958–70 relative to Region V (RR = 0.51, CI 0.40–0.66; p < 0.0001). The difference was most pronounced among women under the age 60 who had 70% lower mortality in Region II than in Region V in the second part of the high exposure period, 1965–70 (RR = 0.30, CI 0.17–0.54; p < 0.0001). Thereafter, mortality from breast cancer remained a little lower in Region II than Region V, but the differences became much smaller, and by the years 2001–2010, the all-ages combined RR was 0.87, CI 0.77–0.99.

Fig. 1 plots the age-adjusted breast cancer mortality RRs comparing Region II with Region V. The arsenic removal plant in Antofagasta commenced operations in 1970 and mortality from breast cancer in Region II started to increase again soon after. In Supplementary Materials p 4–5, we present mortality year by year before, during and after the high exposure period. The Region V breast cancer mortality rates increased gradually over the years, similar to increases reported in many countries (Hermon and Beral, 1996). Although the numbers in any one year are relatively small, the data suggest that breast cancer mortality in Region II started to reduce when the high exposures commenced in 1958. After the arsenic removal plant was installed, mortality rates in Region II gradually increased again until they became close to those in Region V. Since the period of very high exposure in Antofagasta, there have been fluctuations in breast cancer mortality in Region II, with continuation of exposure in many locations. For example, the small town of Chiu Chiu had high concentrations of arsenic in the water (750 μg/L) until the installation of an arsenic removal system in 2001 (Smith et al., 2000, Mac-Lean et al., 2003).

We also examined ovarian cancer mortality; in the high exposure period, the RR reduced to 0.62 (95% CI 0.38–1.02; 2-tailed p = 0.06). Since with ovarian cancer we had a clear a priori hypothesis that mortality might be reduced based on our breast cancer findings, we also calculated a 1-tailed test of significance (p = 0.03). As with breast cancer mortality, the mortality RR was even lower among women aged 30–59 (RR = 0.55, 95% CI 0.29–1.06; 1-tailed p = 0.04). However, the differences between Region II and Region V ovarian cancer mortality were mainly due to a marked increase in Region V (from 3.05 to 6.18 per 100,000), whereas the reduction in Region II rates was relatively small (from 4.82 to 3.52 per 100,000, Supplementary Materials p 6). Reasons for the marked increase in Region V are not known, but data back to 1960 from Greece, Italy and Spain also show similar marked increases in ovarian cancer mortality (Bray et al., 2005).

### 3.1 Treatment of Breast Cancer in Chile

We investigated breast cancer treatment in Chile in the 1950s and 1960s. Research on new breast cancer treatment methods was conducted in University Medical Schools in the capital of Santiago (Lucchini et al., 1962), and not in Region II. Discussions with clinicians in Antofagasta gave no evidence of any special treatment for breast cancer which differed from that in the rest of Chile or Region V. The standard treatment prior to 1970 was radical mastectomy, sometimes followed by radiotherapy. There was no hint of any treatment changes in Region II of Chile in 1958–1970 which could explain our findings.

### 3.2 Breast Cancer Cell Line Studies and Arsenic Concentrations

**Fig. 2:**
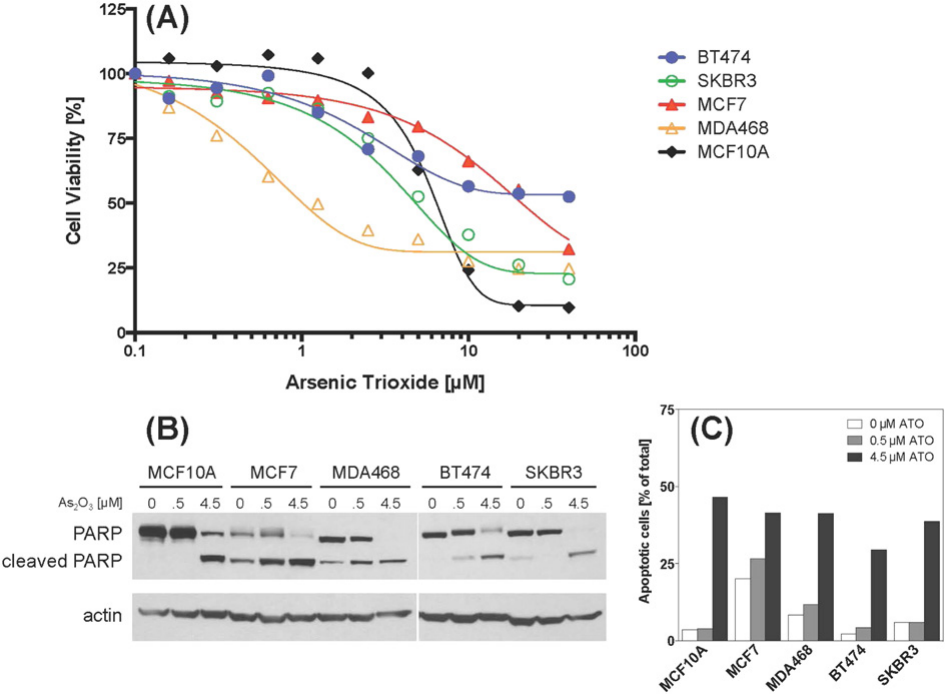
Analysis of the effects of As_2_O_3_ on breast cancer cell lines. MCF10A, MCF7, MDA-468, BT474, and SKBR3 cells were treated with physiologically relevant concentrations of As_2_O_3_ and evaluated at 72 h for cell viability (A), and apoptosis by immunoblotting (B) or Annexin-V staining (C). Panel A: cell viability was assessed by Alamar Blue assay. Results are expressed as the percent of live cells relative to untreated controls. The data represent mean values of three experiments. Panel B: PARP cleavage was detected by immunoblotting and was used as an indication for apoptosis. Actin level was measured as a loading control. Panel C: apoptotic cells were quantified using a Muse™ Annexin V & Dead Cell Assay Kit. Results are expressed as a percentage of total cells.

Fig. 2 (Panel A) presents our findings for human breast cancer cell lines grown up to 72 h in varying concentrations of As_2_O_3_ and assessed by Alamar Blue assay (Supplementary Materials p 1). These cell lines are representative of the major molecular subtypes of breast cancer, including HER2/Neu-positive (SKBR3 and BT474), ER-positive (MCF7), and triple negative/basal-like (MDA-MB-468). Results for the non-tumorigenic immortalized breast epithelial cell line MCF10A are also shown in Fig. 2. In contrast to the breast cancer cells, MCF10A cells demonstrated no reduction in cell viability from As_2_O_3_ treatments at or below 2.5 μM, although viability significantly declined at higher concentrations. BT474, SKBR3, and MCF7 cells demonstrated similar responses to escalating concentrations of As_2_O_3_, with cell viability impaired at the lowest effective doses of 1.25, 2.5, and 2.5 μM, respectively. MDA468 cell viability was significantly impaired at As_2_O_3_ concentrations as low as 0.3 μM. In all cell lines tested, treatment with As_2_O_3_ at concentrations greater than 5 μM produced significant cytotoxic effects.

To investigate the mechanism by which arsenic induces cell cytotoxicity, cell lines were grown for 72 h in As_2_O_3_ (0, 0.5 or 4.5 μM) and apoptosis was examined by immunoblot analysis of PARP cleavage (Fig. 2). In all cell lines tested, treatment with 4.5 μM As_2_O_3_ induced cleavage of endogenous 116 kDa PARP into 85 kDa fragments, indicating the occurrence of apoptotic cell death. Treatment with 0.5 μM As_2_O_3_ induced apoptosis in MCF7, MDA-MB-468, and BT474 cells, but not in SKBR3 or MCF10A cells. Arsenic-induced cell death was also assessed using a Muse™ Annexin V & Dead Cell Assay (Fig. 2, Panel B). Consistent with the immunoblot analysis, Annexin-V staining showed that 72 h of treatment with 4.5 μM As_2_O_3_ produced significant apoptosis in all cell lines tested (Panel C).

The findings from human cancer breast cell lines would indicate that a concentration of 1–2 μM arsenic trioxide is having apoptotic effects with just a few days of exposure. This concentration is close to the human exposure to inorganic arsenic we estimated to have occurred in Antofagasta during the high exposure period (Supplementary Materials p 7).

## 4 Discussion

We have previously reported markedly increased mortality in Region II of Chile from now well established arsenic-related cancers (Marshall et al., 2007, Smith et al., 1998, Steinmaus et al., 2013, Yuan et al., 2010, Ferreccio et al., 2000, Ferreccio et al., 2013). Carcinogenic effects follow arsenic exposure with long latency intervals, usually starting more than ten years after exposure commences, and in the case of lung and bladder cancer, going on even 40 years after exposure reduction (Steinmaus et al., 2013). The reduction in breast cancer mortality in Region II of Chile started quickly after exposure commenced (Supplementary Materials p 4–5), and the trend reversed soon after exposures were reduced in Antofagasta in 1970. The city of Calama, and a number of smaller towns and villages, continued to have high arsenic exposure after 1970, in some cases even up to the year 2000. We believe these exposures may account for the continuing low breast cancer mortality, but we have no satisfactory explanation for the RR reaching about 1 in 1978 and then going down again (Fig. 1). The error bars raise the possibility that there was some random fluctuation in numbers.

The decrease in breast cancer mortality in Region II during the highest exposure period cannot be attributed to chance, with tests of significance yielding p-values less than 0.001. In fact, using the Proc Genmod procedure in SAS for Poisson regression analysis, the test of statistical significance for reduced breast cancer mortality in Region II in the period 1958–1970 yielded a p-value of less than 1 in a million. We therefore focus on whether or not the findings could be attributed to confounding or information bias, and assess biological plausibility.

### 4.1 Confounding

It is inconceivable that findings we present concerning breast cancer could be due to confounding. There are several reasons for this: 1) Risk factors, such as genetics, ethnic composition, age at menarche, and environmental factors such as those related to diet, obesity, smoking and alcohol consumption, could not have changed rapidly enough in the total population of Region II to account for reduced breast cancer mortality compared to Region V starting in 1958, and then reversed themselves rapidly to account for increased mortality starting in 1971; 2) Changes in socioeconomic status and lifestyle are most unlikely to occur in a manner reducing only breast and ovarian cancer mortality, and not mortality from other causes. In contrast to the breast cancer RR of 0.51 (CI 0.40–0.66), the RR for all causes of mortality other than from breast and ovarian cancers in the period 1958–1970 was 0.97 (CI 0.94–0.99) (Table 2); 3) It is most unlikely that confounding could explain reductions in risk of the magnitude we found—for example, the 70% reduction in mortality in women under age 60.

### 4.2 Information Bias

Information bias also cannot explain the findings: 1) Region II is the driest inhabited place on earth and everyone had to drink from the municipal water sources. Exposure misclassification could not bias the findings since virtually everyone in Region II was very highly exposed, and there has never been any evidence of high concentrations of arsenic in water in Region V; 2) Regarding misdiagnosis of cause of death on death certificates, there would have to be a sudden reduction in classifying breast cancer as a cause of death in Region II compared to Region V starting in 1958, and a sudden reversal after 1970. There is no evidence for this, and the mortality differences were most apparent in younger women under the age of 60, when misdiagnosis of breast cancer on death certificates is unlikely. In addition, Chile had (and still has) uniform medical training and health services throughout the country, including Region II and Region V.

### 4.3 Biological Plausibility

Li et al. and Chow et al. studied human breast cancer MCF-7 cells and identified the inhibitory effects of arsenic trioxide on tumor cell proliferation (Li et al., 2004, Chow et al., 2004). They found no cytotoxic effects on normal fibroblast cells at these concentrations and they concluded that arsenic trioxide might be a good candidate for treating breast cancer. Wang et al. reported that arsenic trioxide induces apoptosis in MCF7 cells in a time- and concentration-dependent manner (Wang et al., 2011). In addition, ovarian cancer cell line studies have found them to be responsive to arsenic trioxide (Liu et al., 2012). We assessed the effects of arsenic trioxide on a variety of human cancer cell lines representing major subtypes of breast cancer. In all cell lines tested, As_2_O_3_ treatment suppressed tumor growth starting at concentrations around 1 μM. Our findings are consistent with those previously reported by Xia et al. and Sun et al., both of which demonstrated the cytotoxic effects of micromolar concentrations of arsenic trioxide in multiple breast cancer cell lines (Xia et al., 2012, Sun et al., 2011). Here, we also demonstrated cytotoxic effects on breast cancer cells resulting from submicromolar concentrations of arsenic trioxide, in support of the results of Ahn et al. (2010). We found that submicromolar levels of arsenic trioxide significantly impaired the triple negative breast cancer cell line MDA-MB-468 in a concentration-dependent manner.

The plausibility of our findings in Chile increased further when we considered dose. In vitro assays often involve very high concentrations of toxic agents, much higher than those experienced by humans in daily life. However, the apoptotic effects of As_2_O_3_ on human breast cancer cells start in a few days at doses as low as 1–2 μM (2–4 μM of inorganic arsenic), as seen in Fig. 2 and in previous studies (Xia et al., 2012, Wang et al., 2011, Du et al., 2012, Li et al., 2004, Chow et al., 2004). Women with breast cancer in Antofagasta in the years 1958–1970 would have had continuous exposure with 1–2 μM of inorganic arsenic in their urine (Supplementary Materials p 8), and probably commensurate concentrations in organ tissues throughout their bodies. We are not aware of any data on breast tissue arsenic concentrations, but studies after acute poisoning find arsenic to be distributed in most organs throughout the body (Benramdane et al., 1999). It is also likely that some methylated inorganic arsenic, in particular MMA and DMA in trivalent form, could contribute to breast cancer apoptosis in addition to the activity of inorganic arsenic itself (Zhang et al., 2013).

Ovarian cancer shares with breast cancer several lifestyle and environmental risk factors that are associated with endogenous and exogenous hormone exposure (Zografos et al., 2004), and they also share common genetic risk factors; family history is a risk factor for both. Initially, we had not looked at ovarian cancer mortality in Chile because it is relatively rare. However, when we did so, with a clear a priori hypothesis that the mortality might be reduced, we found that although the numbers were small the trend was similar to that for breast cancer.

We know of no other human data on breast cancer mortality rates associated with *concurrent* exposure to arsenic in drinking water. In addition, as explained in Supplementary Materials p 9, any epidemiological findings concerning arsenic and breast cancer mortality in other populations are unlikely to add significantly to the information we present from Chile.

We suggest that the evidence from Chile, and from in vitro breast cancer cell studies, is sufficient to consider proceeding with a clinical trial of patients with advanced breast cancer. We already have many studies on long-term arsenic health effects, and the side effects associated with treatment of promyelocytic leukemia with arsenic are well-known. Our findings from Chile suggest that high doses sometimes used in treatment of promyelocytic leukemia would not be needed. Intravenous doses of 10 mg of arsenic trioxide per day for leukemia patients result in urine total arsenic concentrations of 4,000 to 5,000 μg/g creatinine in urine (Wang et al., 2013), whereas drinking water arsenic concentrations of about 600 μg/L in our study in Chile resulted in urine total arsenic concentrations of about 600 μg/g of creatinine (Biggs et al., 1997). Importantly, this comparison suggests that inorganic arsenic in drinking water in the treatment of breast cancer could be at much lower doses than those given with intravenous therapy of promyelocytic leukemia.

## 5 Conclusions

We report a major reduction in breast cancer soon after high exposure to inorganic arsenic in drinking water commenced in northern Chile, which could not have resulted from confounding. Once an arsenic removal plant was installed in the major city of Antofagasta, breast cancer mortality started to increase back to rates close to that of the unexposed comparison population. Findings of in vitro studies on human breast cancer give scientific plausibility to these findings. We suggest that there is sufficient supportive evidence to embark on clinical trials of inorganic arsenic in the treatment of advanced human breast cancer.

## Author Contributions

Breast cancer mortality:

AHS, GM, CF, and CS conceptualized and designed the mortality study.

GM and AHS directed mortality data collection and coding of death certificates.

AHS directed mortality data analyses which were conducted by YY.

AHS, JL and YY drafted the paper.

AHS, CS, CF, MTS, YY and JL critically reviewed the manuscript and suggested revisions.

Breast cancer cell line studies:

MDP conceptualized the studies with input from AHS.

MDP directed the laboratory cell line experiments conducted by RMF, MH and LW.

MDP, RMF, MH and LW prepared the text and figures with the cell line study results, which was reviewed by MTS and AHS.

All authors approved the final manuscript for submission.

## Conflicts of Interest

The authors declared no conflicts of interest.

## Funding and Non-financial Support

Funding for this study was provided by the National Institutes of Health (NIH) through grants R01 CA129558 and P42 ES04705. NIH played no role in this study or the preparation of this article. We gratefully acknowledge the advice and information from Alfredo Jadresic, Bruno Nervi, Cesar Sanchez and many other physicians and research scientists in Santiago, Antofagasta and Calama.

## Figures and Tables

**Table 1:** Demographic comparisons of Region II with Region V using available census data for 1952, 1960, and 1970.

	Region II			Region V		
1952	1960	1970	1952	1960	1970
Total population	184,824	215,219	251,906	498,254	617,510	738,336
Urban	89.3%	94.8%	*	85.3%	88.8%	*
Female	46.6%	48.7%	49.7%	52.0%	52.3%	52.5%
Literate	86.0%	92.8%	*	85.5%	91.9%	*
Economically active	54.6%	49.3%	42.8%	51.8%	46.3%	41.9%
Birth rate per 1000	35.0	32.4	24.2	28.2	31.1	22.6
Births to mothers under 20	12.1%	13.2%	*	11.0%	10.6%	*
Births to mothers over 40	3.4%	3.2%	*	4.4%	3.9%	*

*Data not in census.

**Table 2:** Breast and ovarian cancers and all other cause deaths and mortality rate ratios (RRs) comparing Region II in four arsenic exposure periods with Region V which was not exposed to arsenic in drinking water.

Age group	1950–57 low exposure in Region II				1958–70 high exposure in Region II				1971–2000 low-moderate exposure in Region II^a^				2001–2010 low exposure in Region II			
Region		RR	95% CI	Region		RR	95% CI	Region		RR	95% CI	Region		RR	95% CI
II	V			II	V			II	V			II	V		
*Breast cancer*																
30 + total	47	201	1.07	(0.78–1.47)	64	598	0.51	(0.40–0.66)	443	2756	0.76	(0.68–0.83)	280	1437	0.87	(0.77–0.99)
30–59	26	109	1.01	(0.66–1.55)	30	318	0.42	(0.29–0.61)	215	1197	0.72	(0.62–0.83)	138	516	0.95	(0.79–1.15)
60 +	21	92	1.16	(0.72–1.86)	34	280	0.64	(0.45–0.92)	228	1559	0.79	(0.69–0.91)	142	921	0.81	(0.68–0.97)

*Ovarian cancer*																
30 + total	12	32	1.68	(0.87–3.27)	18	137	0.62	(0.38–1.02)	148	593	1.18	(0.99–1.42)	82	399	0.91	(0.72–1.16)
30–59	8	22	1.54	(0.69–3.47)	10	80	0.55	(0.29–1.06)	64	243	1.07	(0.81–1.41)	36	144	0.90	(0.62–1.29)
60 +	4	10	2.03	(0.63–6.47)	8	57	0.74	(0.35–1.55)	84	350	1.28	(1.01–1.63)	46	255	0.92	(0.68–1.27)

*All other causes*																
30 + total	3066	14,995	0.98	(0.94–1.02)	5932	30,732	0.97	(0.94–0.99)	20,249	96,927	1.09	(1.08–1.11)	9968	45,185	1.15	(1.12–1.17)
30–59	1277	5358	1.00	(0.94–1.06)	1993	9218	0.94	(0.90–0.99)	4643	16,726	1.11	(1.07–1.15)	1864	5354	1.23	(1.17–1.30)
60 +	1789	9637	0.96	(0.91–1.01)	3939	21,514	0.98	(0.95–1.01)	15,606	80,201	1.09	(1.07–1.11)	8104	39,831	1.13	(1.11–1.16)

a Excluding 1976 since no data were obtained for that year due to political unrest.

## References

[bb0150] Ahn R.W., Chen F., Chen H. (2010). A novel nanoparticulate formulation of arsenic trioxide with enhanced therapeutic efficacy in a murine model of breast cancer. Clin. Cancer Res..

[bb0005] Antman K.H. (2001). Introduction: the history of arsenic trioxide in cancer therapy. Oncologist.

[bb0155] Benramdane L., Accominotti M., Fanton L., Malicier D., Vallon J.J. (1999). Arsenic speciation in human organs following fatal arsenic trioxide poisoning—a case report. Clin. Chem..

[bb0175] Biggs M.L., Kalman D.A., Moore L.E., Hopenhayn-Rich C., Smith M.T., Smith A.H. (1997). Relationship of urinary arsenic to intake estimates and a biomarker of effect, bladder cell micronuclei. Mutat. Res..

[bb0125] Bray F., Loos A.H., Tognazzo S., La Vecchia C. (2005). Ovarian cancer in Europe: cross-sectional trends in incidence and mortality in 28 countries, 1953–2000. Int. J. Cancer.

[bb0055] Chow S.K., Chan J.Y., Fung K.P. (2004). Inhibition of cell proliferation and the action mechanisms of arsenic trioxide (As_2_O_3_) on human breast cancer cells. J. Cell. Biochem..

[bb0045] Du J., Zhou N., Liu H. (2012). Arsenic induces functional re-expression of estrogen receptor alpha by demethylation of DNA in estrogen receptor-negative human breast cancer. PLoS One.

[bb0095] Ferreccio C., Gonzalez C., Milosavjlevic V., Marshall G., Sancha A.M., Smith A.H. (2000). Lung cancer and arsenic concentrations in drinking water in Chile. Epidemiology.

[bb0135] Ferreccio C., Smith A.H., Duran V. (2013). Case–control study of arsenic in drinking water and kidney cancer in uniquely exposed Northern Chile. Am. J. Epidemiol..

[bb0025] Gallagher R.E. (1998). Arsenic—new life for an old potion. N. Engl. J. Med..

[bb0110] Hermon C., Beral V. (1996). Breast cancer mortality rates are levelling off or beginning to decline in many western countries: analysis of time trends, age-cohort and age-period models of breast cancer mortality in 20 countries. Br. J. Cancer.

[bb0090] International Agency for Research on Cancer (2004). Some Drinking-water Disinfectants and Contaminants, Including Arsenic.

[bb0030] Iriyama N., Yuan B., Yoshino Y. (2013). Aquaporin 9, a promising predictor for the cytocidal effects of arsenic trioxide in acute promyelocytic leukemia cell lines and primary blasts. Oncol. Rep..

[bb0060] Jatoi I., Chen B.E., Anderson W.F., Rosenberg P.S. (2007). Breast cancer mortality trends in the United States according to estrogen receptor status and age at diagnosis. J. Clin. Oncol..

[bb0050] Li X., Ding X., Adrian T.E. (2004). Arsenic trioxide causes redistribution of cell cycle, caspase activation, and GADD expression in human colonic, breast, and pancreatic cancer cells. Cancer Investig..

[bb0140] Liu N., Tai S., Ding B. (2012). Arsenic trioxide synergizes with everolimus (Rad001) to induce cytotoxicity of ovarian cancer cells through increased autophagy and apoptosis. Endocr. Relat. Cancer.

[bb0130] Lucchini A., Arraztoa J., Vargas L. (1962). Metastalysis syndrome and effectiveness of subcutaneous implantation of hexestrol in the treatment of breast cancer metastases. Cancer.

[bb0120] Mac-Lean T., Soto N., Jofre T. (2003). Experiencia de proyecto piloto para abastecer de agua potable a las localidades rurales de lasana y Chiu Chiu. XV Congreso de Ingenieria Sanitaria y Ambiental Aidis—Chile.

[bb0065] Marshall G., Ferreccio C., Yuan Y. (2007). Fifty-year study of lung and bladder cancer mortality in Chile related to arsenic in drinking water. J. Natl. Cancer Inst..

[bb0075] Smith A.H., Goycolea M., Haque R., Biggs M.L. (1998). Marked increase in bladder and lung cancer mortality in a region of Northern Chile due to arsenic in drinking water. Am. J. Epidemiol..

[bb0115] Smith A.H., Arroyo A.P., Guha-Mazumder D.N. (2000). Arsenic-induced skin lesions among Atacameno people in Northern Chile despite good nutrition and centuries of exposure. Environ. Health Perspect..

[bb0105] Smith A.H., Marshall G., Liaw J., Yuan Y., Ferreccio C., Steinmaus C. (2012). Mortality in young adults following in utero and childhood exposure to arsenic in drinking water. Environ. Health Perspect..

[bb0020] Soignet S.L., Maslak P., Wang Z.G. (1998). Complete remission after treatment of acute promyelocytic leukemia with arsenic trioxide. N. Engl. J. Med..

[bb0080] Steinmaus C.M., Ferreccio C., Romo J.A. (2013). Drinking water arsenic in northern chile: high cancer risks 40 years after exposure cessation. Cancer Epidemiol. Biomarkers Prev..

[bb0145] Sun R.C., Board P.H., Blackburn A.C. (2011). Targeting metabolism with arsenic trioxide and dichloroacetate in breast cancer cells. Mol. Cancer.

[bb0100] Venturino P., Ministerio de salud, Servicio de salud Antofagasta, Departamento de programas sobre el ambiente yACdS (1991). Determinacion de concentracion de arsenico urinario en diferentes regiones de Chile. Primera Jornada Sobre Arsenicismo Laboral y Ambiental.

[bb0015] Wang Z.Y., Chen Z. (2008). Acute promyelocytic leukemia: from highly fatal to highly curable. Blood.

[bb0040] Wang Y., Zhang Y., Yang L. (2011). Arsenic trioxide induces the apoptosis of human breast cancer MCF-7 cells through activation of caspase-3 and inhibition of HERG channels. Exp. Ther. Med..

[bb0170] Wang H., Xi S., Liu Z. (2013). Arsenic methylation metabolism and liver injury of acute promyelocytic leukemia patients undergoing arsenic trioxide treatment. Environ. Toxicol..

[bb0070] World Health Organization (2011). Guidelines for Drinking-water Quality.

[bb0035] Xia J., Li Y., Yang Q. (2012). Arsenic trioxide inhibits cell growth and induces apoptosis through inactivation of notch signaling pathway in breast cancer. Int. J. Mol. Sci..

[bb0085] Yuan Y., Marshall G., Ferreccio C. (2010). Kidney cancer mortality: fifty-year latency patterns related to arsenic exposure. Epidemiology.

[bb0160] Zhang Z., Chen Y., Meng H. (2013). Determination of arsenic metabolites in patients with newly diagnosed acute promyelocytic leukemia treated with arsenic trioxide. Leuk. Lymphoma.

[bb0010] Zhou J. (2012). Arsenic trioxide: an ancient drug revived. Chin. Med. J. (Engl.).

[bb0165] Zografos G., Panou M., Panou N. (2004). Common risk factors of breast and ovarian cancer: recent review. Int. J. Gynecol. Cancer.

