# Supplementary material for: Rapid Reduction in Breast Cancer Mortality With Inorganic Arsenic in Drinking Water
Source: eBioMedicine. 2014 Oct 13;1(1):58–63. doi: 10.1016/j.ebiom.2014.10.005 (PMC4286879; doi:10.1016/j.ebiom.2014.10.005)
Supplement: Supplementary file 1 — Supplementary Material. [file mmc1.docx]

**Supplementary Materials**

**Supplementary breast cancer cell study methods**

Cell culture and reagents: Human non-tumorigenic immortalized mammary epithelial MCF10A cells were maintained in MEGM (Lonza) according to the manufacturer’s instructions. Breast cancer cell lines were cultured in RPMI640 medium (MCF7, MDA-MB-468, and SKBR3 cells) or DMEM medium (BT474 cells) supplemented with 10% FBS, 2mM glutamine, and 1% penicillin G-streptomycin solution (Cellgro Mediatech), and incubated in 5% CO2 at 37°C. As_2_O_3_was obtained from Sigma-Aldrich and prepared according to the manufacturer’s instructions.

Alamar Blue cell viability assay*:* For each cell line, 5x103 cells were plated in 96-well plates in 100µl media and incubated overnight in 5% CO2 at 37°C. As_2_O_3_-containing medium was added to a final highest concentration of 40µM with two-fold serial dilutions. Viability was determined after 72h via reduction of alamarBlue (TREK Diagnostic Systems).^1^ Absorbance at 570 and 600 nm was quantified using a Synergy HT plate reader (Biotek).

Immunoblot analysis: Cells were cultured to 80% confluence in 60mm culture dishes in complete growth medium, and then treated with 0, 0·5, or 4·5 µM As_2_O_3_ for 72h. Whole cell lysates were prepared and protein levels were assessed by immunoblot analysis.^2^ Cell lysates were resuspended in sample buffer containing β-mercaptoethanol, boiled 5 min, cooled to RT and loaded onto NuPage (Invitrogen) 7% gels. Separated proteins were transferred to PVDF membranes with an iBlot Dry Blotting System (Invitrogen), then washed, and blocked with 5% milk. Protein expression levels were assessed using anti-PARP 9542 (Cell Signaling) and anti-actin (Abcam) antibodies.

MUSE Annexin-V/7-AAD assay: Apoptotic cells were identified using the Muse™ Annexin V & Dead Cell Assay Kit (Millipore) according to the manufacturer’s instructions. 5x105 cells/well were cultured overnight in 6-well plates in complete growth medium. The next day adherent cells were treated with 0, 0·5, or 4·5 µM As_2_O_3_ and incubated for 72h in 5% CO2 at 37°C. Cells were harvested by trypsination and resuspended in complete growth medium. 100 µl cell suspension were labeled with the same volume of the MuseTM Annexin-V & Dead Cell reagent and incubated for 20 min in the dark at RT. Quantitative detection of Annexin-V/7-AAD positive cells was performed with the MuseTM Cell Analyzer (Millipore). Cells that stained positive for Annexin-V were defined as apoptotic, and were quantified as a percentage of 2000 total events. Positive controls were prepared as follows: cells were treated for 72h with 0, 50, or 450ng/ml cyclohexamide.

**References**

^1^Jegg AM, Ward TM, Irons E, Hoe N, Zhou JY, Liu X, et al. PI3K independent activation of mTORC1 as a target in lapatinib-resistant ERBB2+ breast cancer cells. *Breast Cancer Res Treat* 2012; **136**:683-92.

^2^Iorns E, Ward TM, Dean S, Jegg AM, Thomas D, Murugaesu N, et al. Whole genome in vivo RNAi screening identifies the leukemia inhibitory factor receptor as a novel breast tumor suppressor. *Breast Cancer Res Treat* 2012; **135**:79-91.

**Supplementary table: Sociodemographic characteristics of Region II (Population 493,984 in 2002) and Region V (Population 1,539,852 in 2002*)***

|  | **Region II (%)** | **Region V (%)** | **All of Chile (%)** |
| --- | --- | --- | --- |
| Catholic^a^ | 71·7 | 75·4 |  |
| Urban (vs. rural) ^a^ | 97·7 | 91·6 |  |
| Literacy rate^a^ | 98·2 | 97·0 | 95·8 |
| Education ^a^ |  |  |  |
| No schooling ^a^ | 1·3 | 1·8 | 2·7 |
| University or professional ^a^ | 17·2 | 15·1 | 14·0 |
| Cooking fuel ^a^ |  |  |  |
| Liquid or natural gas ^a^ | 96·4 | 97·5 |  |
| Single family homes ^a^ | 84·8 | 82·4 |  |
| Home appliances ^a^ |  |  |  |
| Refrigerator | 81·9 | 85·8 |  |
| Computer | 27·5 | 20·2 |  |
| Internet connection | 12·0 | 9·4 |  |
| Auto ownership ^a^ | 38·8 | 31·1 |  |
| Smoking^b^ |  |  |  |
| % Non-smokers | 78·0 |  | 77·5 |
| % Moderate smokers* | 21·0 |  | 21·1 |
| % Heavy smokers** | 01·0 |  | 01·2 |
| Hypertension > 140/90 ^c^ | 28·9 | 31·1 | 33·5 |
| Prevalence of morbid obesity^c^ | 2·8 | 0·6 | 1·3 |
| Prevalence of diabetes (total)^c^ | 3·2 | 7·0 | 4·2 |
|  | **Region II (unit)** | **Region V (unit)** | **All of Chile (unit)** |
| Average BMI (kg/cm^2^) ^c^ | 27·6 | 26·8 | 26·8 |
| Average total cholesterol (mg/dl) ^c^ | 174·0 | 185·3 | 186·0 |
| Average serum glucose (mg/dl)^c^ | 85·8 | 94·2 | 92·9 |

* > 0 to 1 pack/day

** > 1 pack/day

^a^Chile census data, <http://www.ine.cl/cd2002/index.php>

^b^Data from the Ministerio de Planificación y Coordinación Nacional República de Chile llla, Encuesta CASEN 1990

^c^Data from Gobierno de Chile, Ministerio de salud. Resultados 1 encuesta de salud, Chile 2003, [http://epi.minsal.cl/epi/html/invest/ENS/InformeFinalENS.pdf](http://epi.minsal.cl/epi/html/invest/ENS/InformeFinalENS.pdf%20)

**Supplementary table:** **Breast cancer deaths and mortality rate ratios (RR) by ten-year age groups comparing Region II in four arsenic exposure periods with Region V which was not exposed to arsenic in drinking water, stratified by age groups**

|  | **1950-57 low exposure**  **in Region II** | | | |  | **1958-70 high exposure**  **in Region II** | | | |  | **1971-2000 low-moderate exposure**  **in Region II*** | | | |  | **2001-2010 low exposure**  **in Region II** | | | |
| --- | --- | --- | --- | --- | --- | --- | --- | --- | --- | --- | --- | --- | --- | --- | --- | --- | --- | --- | --- |
| **Age group** | **Region** | | **RR** | **95% CI** |  | **Region** | | **RR** | **95% CI** |  | **Region** | | **RR** | **95% CI** |  | **Region** | | **RR** | **95% CI** |
|  | **II** | **V** |  |  |  | **II** | **V** |  |  |  | **II** | **V** |  |  |  | **II** | **V** |  |  |
| 30-39 | 4 | 18 | 0·85 | (0·29-2·52) |  | 6 | 47 | 0·48 | (0·21-1·12) |  | 31 | 141 | 0·75 | (0·51-1·10) |  | 18 | 41 | 1·33 | (0·76-2·31) |
| 40-49 | 10 | 43 | 0·97 | (0·49-1·92) |  | 9 | 98 | 0·39 | (0·20-0·78) |  | 84 | 460 | 0·69 | (0·54-0·87) |  | 45 | 200 | 0·76 | (0·55-1·05) |
| 50-59 | 12 | 48 | 1·12 | (0·60-2·11) |  | 15 | 173 | 0·41 | (0·24-0·70) |  | 100 | 596 | 0·73 | (0·59-0·91) |  | 75 | 275 | 1·03 | (0·80-1·33) |
| 60-69 | 12 | 42 | 1·34 | (0·70-2·54) |  | 17 | 136 | 0·64 | (0·39-1·06) |  | 99 | 643 | 0·78 | (0·63-0·97) |  | 41 | 312 | 0·61 | (0·44-0·84) |
| 70-79 | 6 | 30 | 1·04 | (0·44-2·51) |  | 10 | 87 | 0·60 | (0·31-1·15) |  | 81 | 533 | 0·85 | (0·67-1·07) |  | 58 | 309 | 1·00 | (0·76-1·32) |
| 80+ | 3 | 20 | 0·89 | (0·26-2·98) |  | 7 | 57 | 0·72 | (0·33-1·58) |  | 48 | 383 | 0·74 | (0·55-0·99) |  | 43 | 300 | 0·87 | (0·63-1·20) |
| **30+ Total** | 47 | 201 | 1·07 | (0·78-1·47) |  | 64 | 598 | 0·51 | (0·40-0·66) |  | 443 | 2756 | 0·76 | (0·68-0·83) |  | 280 | 1437 | 0·87 | (0·77-0·99) |

* excluding 1976 since no data were obtained for that year due to political unrest

**Supplementary table: Breast cancer deaths by year, rates in Regions II and V and age-adjusted**

**Rate Ratios with the high exposure period 1958-1970 in bold**

| **Year** | **Region II deaths** | **Region V deaths** | **Region II**  **rate** | **Region V**  **rate** | **Rate Ratio** |
| --- | --- | --- | --- | --- | --- |
| 1950 | 2 | 21 | 6·97 | 17·28 | 0·44 |
| 1951 | 2 | 27 | 6·85 | 21·89 | 0·33 |
| 1952 | 8 | 21 | 26·91 | 16·79 | 1·73 |
| 1953 | 5 | 23 | 16·34 | 17·87 | 1·01 |
| 1954 | 8 | 26 | 25·44 | 19·64 | 1·42 |
| 1955 | 10 | 35 | 30·95 | 25·73 | 1·31 |
| 1956 | 5 | 27 | 15·07 | 19·33 | 0·82 |
| 1957 | 7 | 21 | 20·57 | 14·65 | 1·58 |
| **1958** | **4** | **27** | **11·46** | **18·37** | **0·69** |
| **1959** | **7** | **34** | **19·58** | **22·57** | **0·94** |
| **1960** | **5** | **38** | **13·65** | **24·62** | **0·58** |
| **1961** | **5** | **39** | **13·40** | **24·62** | **0·60** |
| **1962** | **6** | **37** | **15·78** | **22·78** | **0·78** |
| **1963** | **7** | **41** | **18·08** | **24·62** | **0·81** |
| **1964** | **5** | **43** | **12·68** | **25·21** | **0·55** |
| **1965** | **5** | **46** | **12·46** | **26·34** | **0·53** |
| **1966** | **2** | **69** | **4·90** | **38·61** | **0·14** |
| **1967** | **3** | **49** | **7·23** | **26·81** | **0·29** |
| **1968** | **8** | **64** | **18·95** | **34·26** | **0·62** |
| **1969** | **4** | **44** | **9·32** | **23·05** | **0·46** |
| **1970** | **3** | **67** | **6·88** | **34·37** | **0·22** |
| 1971 | 6 | 67 | 13·28 | 33·40 | 0·44 |
| 1972 | 3 | 57 | 6·42 | 27·64 | 0·25 |
| 1973 | 11 | 49 | 22·79 | 23·12 | 1·13 |
| 1974 | 6 | 52 | 12·04 | 23·90 | 0·56 |
| 1975 | 11 | 67 | 21·42 | 30·02 | 0·82 |
| 1976 | No data are available for 1976 for anywhere in Chile | | | | |
| 1977 | 13 | 59 | 23·87 | 25·16 | 1·06 |
| 1978 | 11 | 85 | 19·64 | 35·39 | 0·64 |
| 1979 | 16 | 82 | 27·80 | 33·36 | 0·97 |
| 1980 | 21 | 73 | 35·53 | 29·03 | 1·39 |
| 1981 | 9 | 72 | 14·84 | 28·00 | 0·60 |
| 1982 | 13 | 90 | 20·90 | 34·25 | 0·71 |
| 1983 | 7 | 78 | 10·83 | 28·84 | 0·43 |
| 1984 | 14 | 98 | 20·89 | 35·23 | 0·69 |
| 1985 | 14 | 108 | 20·17 | 37·78 | 0·62 |
| 1986 | 16 | 96 | 22·27 | 32·71 | 0·79 |
| 1987 | 13 | 108 | 17·51 | 35·86 | 0·57 |
| 1988 | 17 | 98 | 22·18 | 31·73 | 0·79 |
| 1989 | 12 | 104 | 15·18 | 32·85 | 0·54 |
| 1990 | 17 | 98 | 20·87 | 30·22 | 0·82 |
| 1991 | 21 | 118 | 25·04 | 35·55 | 0·82 |
| 1992 | 16 | 110 | 18·55 | 32·39 | 0·67 |
| 1993 | 15 | 112 | 16·87 | 32·19 | 0·59 |
| 1994 | 20 | 118 | 21·84 | 33·13 | 0·77 |
| 1995 | 14 | 135 | 14·86 | 37·04 | 0·49 |
| 1996 | 27 | 121 | 27·87 | 32·46 | 1·00 |
| 1997 | 23 | 118 | 23·11 | 30·97 | 0·89 |
| 1998 | 28 | 142 | 27·40 | 36·47 | 0·91 |
| 1999 | 21 | 109 | 20·03 | 27·41 | 0·87 |
| 2000 | 28 | 132 | 26·04 | 32·52 | 0·97 |
| 2001 | 26 | 115 | 23·60 | 27·77 | 1·01 |
| 2002 | 31 | 126 | 27·48 | 29·83 | 1·10 |
| 2003 | 29 | 129 | 25·06 | 29·86 | 0·98 |
| 2004 | 31 | 125 | 26·13 | 28·31 | 1·10 |
| 2005 | 25 | 136 | 20·57 | 30·14 | 0·80 |
| 2006 | 23 | 157 | 18·48 | 34·07 | 0·66 |
| 2007 | 26 | 155 | 20·41 | 32·95 | 0·75 |
| 2008 | 27 | 168 | 20·73 | 35·00 | 0·72 |
| 2009 | 31 | 161 | 23·28 | 32·89 | 0·89 |
| 2010 | 31 | 165 | 22·78 | 33·06 | 0·85 |

Note: We have no explanation for the low mortality in years 1950 and 1951, and the reduced RRs could be due to chance (p>0·05). As presented in Table 2 of the main paper, the RR estimate for the whole pre-exposure period 1950-1957 was 1·07 (0·78-1·47).

**Supplementary Table: Ovarian cancer deaths by four arsenic exposure periods, rates in Regions II and V and age-adjusted Rate Ratios (RR) with the high exposure period 1958-1970 in bold**

| **Year** | **Region II deaths** | **Region V deaths** | **Region II**  **rate** | **Region V**  **rate** | **Rate Ratio** |
| --- | --- | --- | --- | --- | --- |
| 1950-1957 | 12 | 32 | 4·82 | 3·05 | 1·68 |
| **1958-1970** | **18** | **137** | **3·52** | **6·18** | **0·62** |
| 1971-2000* | 148 | 593 | 6·94 | 6·88 | 1·18 |
| 2001-2010 | 82 | 399 | 6·67 | 8·75 | 0·91 |

* excluding 1976 since no data were obtained for that year due to political unrest

**Supplementary Table: Breast cancer cell viability with exposure to arsenic trioxide for 72 hours**

| **ATO [μM]** | **MCF10A** | **MCF7** | **MDA468** | **BT474** | **SKBR3** |
| --- | --- | --- | --- | --- | --- |
| 0·1 | 100·00 | 100·00 | 100·00 | 100·00 | 100·00 |
| 0·16 | 105·85 | 97·17 | 86·92 | 90·43 | 91·24 |
| 0·31 | 102·91 | 92·65 | 76·10 | 94·48 | 89·31 |
| 0·63 | 107·23 | 90·59 | 60·29 | 99·17 | 92·53 |
| 1·25 | 105·85 | 89·67 | 49·76 | 85·04 | 86·48 |
| 2·5 | 100·24 | 83·24 | 39·57 | 70·83 | 75·07 |
| 5 | 62·86 | 79·56 | 36·06 | 68·13 | 52·55 |
| 10 | 24·19 | 66·23 | 27·71 | 56·50 | 37·85 |
| 20 | 10·31 | 55·18 | 24·80 | 53·65 | 26·25 |
| 40 | 9·74 | 32·29 | 24·87 | 52·46 | 20·67 |

**Estimation of arsenic concentrations for exposed women in Chile**

Arsenic ingested in drinking water is mainly excreted in the urine (about 70%).^1^ In the north of Chile, each city and town had only one water source, since this region is the driest inhabited place on earth and there were no private wells or other sources of drinking water. Between 1992 and 1994, we studied residents in the town of San Pedro de Atacama who were drinking water containing arsenic concentrations of around 600 µg/L, and measured urine concentrations of arsenic in 124 town residents.^1,2^ A few years later, we undertook a study in the small town of Chiu Chiu in Region II and obtained urine arsenic measurements related to a town water arsenic concentrations of around 750 µg/L.^3^ Based on these studies, we estimated urine concentrations of inorganic arsenic for drinking water concentrations experienced by the residents of Antofagasta from 1958-1970.

In our study in the town of San Pedro de Atacama, their drinking water arsenic concentration at the time was about 600 µg/L.^1^ The average urine arsenic concentration including inorganic arsenic and its two major metabolites (MMA and DMA) was 582 µg/L. The fraction that remained in the inorganic form (i.e., subtracting out MMA and DMA) was 107 µg/L (or about 1·4 µM). Non-inorganic species in urine were mainly inorganic arsenic metabolites MMA (monomethylated arsenic) and DMA (dimethylated arsenic). One can infer from this study that in Antofagasta, when the water concentrations were about 870 µg/L, urinary concentrations of inorganic arsenic would probably have been in the 1-2 µM range (75 to 150 µg/L).

In 1999, we conducted a small study in the town of Chiu Chiu in Region II when their water arsenic concentrations were around 750 µg/L.^3^ The average urine arsenic concentration among women in the study was 466 µg/L, and the percentage in inorganic form was 15%. These data also support that women in Antofagasta at the time of the high exposure would have had urinary concentrations of inorganic arsenic in the 1-2 µM range.

**References**

^1^Biggs ML, Kalman DA, Moore LE, Hopenhayn-Rich C, Smith MT, Smith AH. Relationship of urinary arsenic to intake estimates and a biomarker of effect, bladder cell micronuclei. *Mutat Res* 1997; **386**(3):185-95.

^2^Hopenhayn-Rich C, Biggs ML, Smith AH, Kalman DA, Moore LE. Methylation study of a population environmentally exposed to arsenic in drinking water. *Environ Health Perspect* 1996; **104**(6):620-8.

^3^Smith AH, Arroyo AP, Mazumder DN, Kosnett MJ, Hernandez AL, Beeris M, et al. Arsenic-induced skin lesions among Atacameno people in Northern Chile despite good nutrition and centuries of exposure. *Environ Health Perspect* 2000; **108**(7):617-20.

**Further significant epidemiological evidence is unlikely to be found**

We know of no other existing human evidence of breast cancer mortality rates associated with concurrent exposure to arsenic in drinking water. Two studies report breast cancer mortality in populations with past exposure to arsenic. In an ecologic study in a highly arsenic exposed area in Taiwan, decreased breast cancer standardized mortality ratios (SMRs) were reported compared to national mortality rates (SMR=0·67, 95% CI 0·49-0·89), but not compared to a more valid local reference population (SMR=1·01, 95% CI 0·74-1·34).^1^ Also, the authors state that during 1956 and thereafter a tap water system was gradually installed and mortality in this study was assessed from 1971 to 1994, so this study actually has no mortality information at or near the time of the high arsenic exposure.

A study in a Mormon cohort in the United States at relatively low arsenic exposure also did not report mortality with concurrent exposure. Breast cancer mortality in the cohort was reduced compared to all of Utah (SMR=0·58, 95% CI 0·36-0·88) but there was no evidence of a dose-response relationship with arsenic within the Mormon cohort, since those with the lowest arsenic exposure had an SMR of 0·64.^2^ This suggests that the breast cancer mortality reduction in the Mormon cohort was not related to arsenic in drinking water.

It is possible that studies in other arsenic-exposed populations in the world could find further evidence but they would require large studies assessing breast cancer mortality concurrent with high concentrations of arsenic in drinking water. Cohort studies in Bangladesh in arsenic-exposed population could examine breast cancer mortality. However, the largest cohort study conducted there involved 115,000 participants but had only about 9% (approximately 10,000 people) with historic arsenic exposure above 300 µg/L.^3^ This contrasts with the city of Antofagasta in Chile, in which 125,000 people had arsenic in their water for 12 years at concentrations over 800 µg/L, and with the total population of Region II of over 250,000 the large majority of whom were exposed to arsenic in water. This contrast gives emphasis to the uniqueness of the arsenic exposure in Region II of Chile, and indicates that any epidemiological findings concerning arsenic and breast cancer mortality in other populations are unlikely to add significantly to the information we present from Chile.

**References**

^1^ Tsai SM, Wang TN, Ko YC. Mortality for certain diseases in areas with high levels of arsenic in drinking water. *Arch Environ Health* 1999; **54**(3):186-93.

^2^ Lewis DR, Southwick JW, Ouellet-Hellstrom R, Rench J, Calderon RL. Drinking water arsenic in Utah: a cohort mortality study. *Environ Health Perspect* 1999; **107**(5):359-65.

^3^ Sohel N, Persson LA, Rahman M, Streatfield PK, Yunus M, Ekstrom EC, et al. Arsenic in drinking water and adult mortality: a population-based cohort study in rural Bangladesh. *Epidemiology* 2009; **20**(6):824-30.
